# Genetic Effects on Dispersion in Urinary Albumin and Creatinine in Three House Mouse (*Mus musculus*) Cohorts

**DOI:** 10.1534/g3.118.200940

**Published:** 2019-01-03

**Authors:** Guy M. L. Perry

**Affiliations:** Department of Biology, University of Prince Edward Island, Charlottetown, PEI, Canada C1A 4P3

**Keywords:** phenotypic dispersion, albuminuria, creatinine, mouse, *Mus musculus*, genetic homeostasis, negative dominance

## Abstract

Conventionally, quantitative genetics concerns the heredity of trait means, but there is growing evidence for the existence of architectures in which certain alleles cause random variance in phenotype, termed ‘phenotypic dispersion’ (*PD*) or ‘variance QTL’ (vQTL), including in physiological traits like disease signs. However, the structure of this phenomenon is still poorly known. *PD* for urinary albumin (*PD_UAlb_*) and creatinine (*PD_UCrea_*) was mapped using curated data from two nearly genetically identical F_2_ mouse (*Mus musculus*) cohorts (383 male F_2_ C57BL/6J×A/J (97 SNP) and 207 male F_2_ C57BL/6J×A/J *ApoE* knockout mice (144 SNP)) and a related mapping cohort (340 male F_2_ DBA/2J×C57BL/6J (83 SNP, 8 microsatellites)). *PD_UAlb_* was associated with markers in regions of Chr 1 (5-64 megabases (MB); 141-158 MB), 3 (∼113 MB), 8 (37-68 MB), 14 (92-117 MB) and 17 (14-24 MB) with several positions and quantitative architectures in common between the two C57BL/6J×A/J cohorts, most of which had a negative dominant construction. One locus for *PD_UCrea_* was detected on Chr 19 (57 MB) in the C57BL/6J×A/J *ApoE*^−/−^ cohort. The large number of negative dominant loci for albuminuria dispersion relative to conventional quantitative trait loci suggests that the development of albuminuria may be largely genetically dynamic and that randomization in this development is detrimental.

Conventional quantitative genetics concerns heritable differences in mean phenotype (Roff 1997). However, there is increasing evidence that some genotypes confer significant differences in random or residual variability rather than stable mean phenotype, so that intra-individual or inter-individual randomization among genotypes or genetic groups may constitute a properly heritable genetic effect instead of sheer error (Reeve and Robertson 1953; Perry *et al.* 2003; Sorensen and Waagepetersen 2003; Ordas *et al.* 2008; Rönnegård and Valdar 2012). This effect has been described as ‘*phenotypic dispersion*’ (*PD*) (Perry *et al.* 2012a) and may reflect the effects of ‘variance QTL’ (vQTL) on trait variance (Rönnegård and Valdar 2012). As early as the 1950s, divergent selection experiments in *Drosophila* found simultaneous changes in means and variances for wing length and body size (Reeve and Robertson 1953; Clayton and Robertson 1957), suggesting the accumulation of both alternate variants and randomizing alleles via incidental inclusion of extreme individuals during selection (Hill and Zhang 2004). Since that point, genetic variation for heterogeneity has been found in plants (Hall *et al.* 2007; Ordas *et al.* 2008), fish (Perry *et al.* 2003), birds (Rowe *et al.* 2006; Wolc *et al.* 2009) and mammals (SanCristobal-Gaudy *et al.* 1998; Sorensen and Waagepetersen 2003; Rönnegård *et al.* 2010; Perry *et al.* 2012a), including rodent disease models (Ibáñez-Escriche *et al.* 2008) and human phenotypes and gene expression (Perry *et al.* 2012c; Hulse and Cai 2013; Perry *et al.* 2013). Theoretical investigations of residual variance suggest a genetic architecture resembling classical trait means (*μ*_i_, *σ*_i_) (Hill and Zhang 2004; Hill and Mulder 2010) or the general inability of inbred individuals to buffer minor environmental perturbation (Lerner 1977). Most examples of dispersion come from common environments (*i.e.*, Perry *et al.* 2003; Sorensen and Waagepetersen 2003; Wolc *et al.* 2009; Rönnegård *et al.* 2010; Perry *et al.* 2012a; Sell-Kubiak *et al.* 2015; Conley *et al.* 2018) so that an explanation of heredity for environmental buffering (de Visser *et al.* 2003) seems improbable, although an assay of dispersion in airway hyperresponsiveness (AHR) found increasing genotypic differences in *PD* at a Chr 10 locus with increasing methacholine dosage, suggesting environmental gradients in the expression of dispersion loci (G. M. L. Perry, unpublished data). Little, however, is known of wider trends in the quantitative construction of dispersive systems, so that the relative contributions of additivity and dominance to this phenomenon and their meaning (*c.f*. Roff 1997) are not understood. A strong additive basis, for example, would indicate the functional independence of individual alleles within genotypes.

Disease physiology appears susceptible to the dispersive/vQTL effect (Perry *et al.* 2012a; Perry *et al.* 2013). In this work, genome-wide associations of single nucleotide polymorphism (SNP) genotype with dispersion in urinary albumin, urinary creatinine and blood urea nitrogen were tested using curated data from three mouse (*Mus musculus*) groups, consisting of i) a cohort of F_2_ intercrosses of albuminuric A/J mice with non-albuminuric C57BL/6Js, ii) a cohort of F_2_ A/J × C57BL/6J *ApoE^−/−^* knockout mice (Doorenbos *et al.* 2008) and iii) an F_2_ intercross of C57BL/6J×DBA/2J mice (Sheehan *et al.* 2007). This data were originally used to scan for standard loci affecting albumin excretion, an early indicator of chronic kidney disease (CKD) and diabetic nephropathy resulting from podocyte damage and immune cell recruitment, and to determine the genetic role of *Apoe* in albuminuria (Joss *et al.* 2005; Doorenbos *et al.* 2008; Coto *et al.* 2013), and down-regulates mesangial cell proliferation associated with renal disease (Chen *et al.* 2001).

Several genomic regions were significantly associated with phenotypic dispersion in urinary albumin ($PD_{UAlb}$) in these cohorts after correction at the 5% False Discovery Rate (FDR), including Chr 1 (5-64 and 141-158 MB (termed $PD_{UAlb}1$ and $PD_{UAlb}2$, respectively)), 3 (∼125 MB ($PD_{UAlb}3$)), 8 (37-68 MB ($PD_{UAlb}4$)), 14 (92-117 MB ($PD_{UAlb}5$)) and 17 (14-24 MB ($PD_{UAlb}6$)). A single marker on chromosome 19 (19-060823449-M, 56.5 MB ($PD_{UCrea}1$)) was associated with dispersion in urinary creatinine. Notably, a clear majority of albuminuria dispersion loci were at least partially negative-dominant in this assay, suggesting that random variance in disease physiology may be largely detrimental, or integral to the process of disease itself. These findings do not agree with the expectation that ubiquitous physiological systems underlie dispersion, but do support the case that phenotypic dispersion is physiologically relatively common and indicate a major jump in the understanding of the overall meaning of the effect to physiology and survivorship.

## Materials and Methods

### Cohorts 1, 2 (Doorenbos *et al.* 2008)

All information in this study was derived from archived collections hosted with and curated by The Jackson Laboratory in the Churchill Group QTL Archive (https://phenome.jax.org/centers/QTLA). The first two sets (see Doorenbos *et al.* 2008; MPD:208) were derived from albuminuric A/J, normouric C57BL/6J (B6) and normouric B6-129P2-*ApoE*^tm1Unc^/J (B6 *Apoe*^−/−^) progenitor mice (an *Apoe* knockout backcrossed into the B6 line for 12 generations) were obtained from the Jackson Laboratories. B6 males and A/J females were bred to create B6×A/J F_1_s, which were bred in turn to create 383 F_2_ C57BL/6J×A/J intercrosses (‘Doorenbos *et al.* A’). B6 *Apoe*^−/−^ males and A/J females were used to breed B6 *Apoe*^−/−^×A/J hybrids which were used in turn to create 207 male F_2_ homozygous *ApoE* knockout B6 *Apoe*^−/−^×A/J mice (‘Doorenbos *et al.* B’) (Doorenbos *et al.* 2008).

Spot urine samples from each mouse were quantified for urinary creatinine (UCrea; mg/dl) as an estimate of baseline kidney function/glomerular throughput and albumin (UAlb; mg/dl). Weight (g) and blood urea nitrogen (BUN; mg/dl) were available in Doorenbos *et al.* B but not in Doorenbos *et al.* A or Sheehan *et al.* Genomic DNA was isolated as per Korstanje *et al.* (2004). Ninety-seven single nucleotide polymorphisms (SNP) with a roughly even distribution across all autosomes and the X chromosome were genotyped in Doorenbos *et al.* A, and 144 in Doorenbos *et al.* B.

### Cohort 3 (Sheehan *et al.* 2007)

The third cohort (Sheehan *et al.* 2007; MPD:205), consisting of male F_2_ C57BL/6J (B6) × DBA/2J (D2) mice F_1_ phenotyped for urinary creatinine and albumin and reported in Sheehan *et al.* (2007). This cohort shared only one of the source strains with the above two cohorts (DBA/2J) and was included for comparison to those more closely related groups. F_1_ reciprocal families ([B6×D2] and [D2×B6]) were bred from B6 and D2 mice from the Jackson Laboratories. An additional F_1_ [D2×B6] cohort was produced and used to breed a total of 340 F_2_ C57BL/6J×DBA/2J [‘BxD’] male mice from an initial cross of B6 females bred with DBA2 males. F_2_ mice were phenotyped with spot urine collections and genotyped over all 19 autosomes and the X chromosome using 83 SNP, and at eight microsatellites on chromosome 2, for a mean intermarker spacing of 17 cM.

Marker location was assigned throughout based on the Cox *et al.* (2009) reference marker map build using base-pair (BP) distances to avoid possible mis-position from sex differences in recombination by region (Broman *et al.* 1998; Sakamoto *et al.* 2000; Popa *et al.* 2012).

#### Animal usage:

Ethical animal use in the original studies was monitored and approved by the Institutional Animal Care and Use Committee (IACUC) of The Jackson Laboratory.

#### Association analysis:

All analysis was performed in SAS (2011). In order to protect against distributional errors, the deviation of individuals from predicted multivariate values were estimated from externally Studentized residuals (Steel and Torrie 1980) in which ordinary residuals *ε* are divided by their standard errors (${\hat{\varepsilon }}_{s}=\hat{\varepsilon }/(\hat{\sigma }\sqrt{1-h_{ii}}$) (where $h_{ii}$ is the observation leverage and ${\hat{\sigma }}_{\left(i\right)}^{2}=\sum_{\begin{array}{l} j=1\\ j\neq i \end{array}}^{n}{\hat{\varepsilon }}_{j}^{2}/(n-m-1)$), in order to satisfy the experiment-wise relations $\sum_{i=1}^{n}{\hat{\varepsilon }}_{i}=0$ and $\sum_{i=1}^{n}{\hat{\varepsilon }}_{i}x_{i}=0$. Individual Studentized residuals were estimated in a general linear model of the form

$$y_{ij}=\mu +\alpha_{i}+\beta_{MLH}X_{MLH}+\beta_{Ucrea}X_{Ucrea}+\varepsilon_{ij}$$

where $y_{ij}$ is albuminuria or creatinine for individual *j*,μ is the mean phenotype for the cohort, $\alpha_{i}$ is the effect of marker locus *i*, $\beta_{MLH}X_{MLH}$ is the partial regression effect of multilocus heterozygosity (MLH), $\beta_{Ucrea}X_{Ucrea}$ is the partial regression effect for glomerular filtration rate and $\varepsilon_{ij}$ is individual residual error. MLH was included at this level to account for possible inbreeding effects and calculated as MLH = *n_het_*/*n_total_* within individuals in each group across all available genotypes. Regression effects for creatinine were only included for albuminuria. Each model was initially run without locus terms at each analytical stage order to determine covariates for the genomic models including locus terms, which were used in order to account for the effects of known and undetected conventional loci on albuminuria (see Doorenbos *et al.* 2008).

Individual residual error estimates (${\hat{\varepsilon }}_{ij}$) were absolute-transformed (|${\hat{\varepsilon }}_{ij}$|); as absolute divergence of any particular individual from that predicted by genotype, these were then considered to be phenotypic dispersion (PD) for that trait ($PD_{UAlb}$, $PD_{UCrea}$). Since absolute-transformed distributions are left-skewed with strong lower bounds at the abscissa, marker-dispersion associations were fit using Tobit quantitative and limited models (Tobin 1958) with a lower bound of zero with PD as the dependent variable and locus as an independent variable along with significant covariates. In Tobit censored distributions, the actual y of the true variable y* is only observed where y > τ, the lower truncation value, and as y* otherwise (*i.e.*, y = y* where y > τ). The truncated PDF of such a system then is expressed as f(y|y>τ)=f(y)((P(y>τ)) and transforming by { a=−μ/σ,b=1/σ} produces $P(y>\tau )=1-\Phi \left(\frac{\tau -\mu }{\sigma }\right)=1-\Phi (\alpha )$, where $\alpha =\frac{\tau -\mu }{\sigma }$, and $\Phi \left(\frac{\tau -\mu }{\sigma }\right)$ is the cumulative distribution function (CDF; Steel and Torrie 1980; Greene 2002) of the original data so that the likelihood becomes

$$L=\prod_{i=1}^{N}\frac{f(y)}{1-\Phi (\alpha )}.$$

Model terms were optimized by the default quasi-Newtonian Broyden-Fletcher-Goldfarb-Shanno algorithm (Press *et al.* 2007). The significance of genotypic effects in the analysis of each locus was determined via a joint nonequivalence Wald contrast against mean *PD* in the referential A/J homozygote $\left(H’=\langle \mu_{PD_{i}}^{CC}=0,\;\mu_{PD_{i}}^{CA}=0\rangle \right)$ (see Parsad 2008; SAS 2014), the last genotype being fit via default as the referential genotype against the rest of the population. MLH was included as a covariate where it was significantly associated with *PD* (*P* < 0.1) to account for the possible production of phenoaberrancy by the failure of increasingly inbred individuals to buffer phenotype against exogenous and endogenous stresses, termed *genetic homeostasis* (Lerner 1977). Additivity and dominance were estimated in SAS using contrast statements equivalent to Griffing’s potence ratio $h_{P}=\left(2\mu_{CA}-\left(\mu_{CC}+\mu_{AA}\right)\right)/\left(\mu_{CC}-\mu_{AA}\right)=Q/L$ (Griffing 1990) where *Q* is the quadratic dominant effect and *L* is the classical linear differentiation between alternate homozygotes. Additivity was tested by contrast against the midparent phenotype $\left(\left(PD_{C}+PD_{A}\right)/2\right)$ (contrast statement +1 0 -1). Dominance was tested sequentially using the vectors $Q=[+0.5_{CC}+0.5_{CA}-1.0_{AA}]$ and $Q=[-1.0_{CC}+0.5_{CA}+0.5_{AA}]$ to test positive dominance and $Q=[+1.0_{CC}-0.5_{CA}-0.5_{AA}]$ and $Q=[-0.5_{CC}-0.5_{CA}+1.0_{AA}]$ to test negative dominance (Aurelio *et al.* 2000; Lee and Sabapathy 2008).

#### Significance:

Significance thresholds were adjusted via Benjamini-Hochberg (Verhoeven *et al.* 2005) by trait calculated across all markers independently without reference to linkage among markers at the classic False Discovery Rate (*P* ≤ *k*_i_*α/*m*) with a ‘hard floor’ for rejection of H’ at a nominal *P*_i_ = 0.01.

#### SNP sites for QTL for albumin excretion:

The Mouse Genome Informatics (MGI) resource curated by the Jackson Laboratories (www.informatics.jax.org) was used to identify SNP between the source strains (C57BL/6J *vs.* A/J; DBA/2J *vs.* C57BL/6J) at nonsynonymous coding sites (CNS), utranslated mRNA sequence (mRNA-UTR), splice sites (SS) and non-coding transcript variants (NTV) (see Ward and Kellis 2012) at genes closely linked (<10 MB) to consensus markers for $PD_{Ualb}$ as possible candidates for genetic effects on dispersion. Sequence information was based on the dbSNP (Mouse) Build 142 by MGI and the GRCm38 mouse genomic build. Annotation functions were obtained through databases from The Jackson Laboratories (www.informatics.jax.org), the European Bioinformatics Institute (www.ebi.ac.uk), UniProt (www.uniprot.org), GeneCards (www.genecards.org), WikiGenes (www.wikigenes.org) and homologs listed with the Rat Genome Database (www.rgd.mcw.edu).

### Data availability

All data used in this work were archived and curated by the Churchill Group QTL Archive, Jackson Laboratory, Bar Harbor, MA, USA (https://phenome.jax.org/centers/QTLA) (IDs: MPD: 205, 208). Supplemental material available at Figshare: https://doi.org/10.25387/g3.6853580.

## Results

### Multilocus heterozygosity

MLH was negatively correlated with UAlb in Doorenbos *et al.* A (*β* = -4.87 (SE 1.14), *P* < 0.0001) and marginally negatively correlated with UAlb in Doorenbos *et al.* B (*β* = -3.76 (SE 2.02), *P* = 0.0650). MLH was also negatively associated with $PD_{UAlb}$ in Doorenbos *et al.* A (*β* = -1.72 (SE 0.390), *P* < 0.0001) and B (*β* = -2.29 (SE 0.884), *P* = 0.0104). MLH was not associated with UAlb (*P* > 0.6) or $PD_{UAlb}$ (*P* > 0.6) in Sheehan *et al.*

UAlb was significantly positively correlated with UCrea in Doorenbos *et al.* A (*β* = 0.0130 (SE 0.00593), *P* = 0.0287), Doorenbos *et al.* B (*β* = 0.0113 (SE 0.00603), *P* = 0.0630) and Sheehan *et al.* (*β* = 0.0218 (SE 0.00380), *P* < 0.0001). $PD_{UAlb}$ was positively associated with UCrea in Doorenbos *et al.* A (*β* = 0.00544 (SE 0.00203), *P* = 0.0077) and B (*β* = 0.00680 (SE 0.00264), *P* = 0.0110) and in Sheehan *et al.* (*β* = 0.0118 (SE 0.00203), *P* < 0.0001).

### Association analysis

Doorenbos *et al.* A covered a linkage distance of 1.22 M, Doorenbos *et al.* B 1.18 M and Sheehan *et al.* 1.17 M. A total of 97 SNP markers were available for Doorenbos *et al.* A, 144 SNP markers for Doorenbos *et al.* B, and eight microsatellites and 83 SNP in Sheehan *et al.* (Table 1).

**Table 1:** Marker proportions, mapping completeness and record completeness for 1) 383 male F_2_ C57BL/6J × A/J mice (Doorenbos *et al.* A group), 2) 207 male F_2_ C57BL/6J-*Apoe^−/−^* mice (Doorenbos *et al.* B group) (Doorenbos *et al.* 2008) and 3) 340 male F_2_ DBA/2J×C57BL/6J mice (Sheehan *et al.* 2007). N refers to the number of records available for each phenotype (urinary albumin, blood urea nitrogen (BUN) and creatinine), *µ* is the cohort mean for that phenotype and Range the min-max range for all observations

	Albumin			BUN			Creatinine		
Cohort	n	*µ*	Range	N	*µ*	Range	n	*µ*	Range
Doorenbos *et al.* A	383	0.72	0-35	—	—	—	382	76.1	29-150
Doorenbos *et al.* B	80	0.85	0-15	131	20.4	14-30	152	78.6	0-182.7
Sheehan	340	0.96	0-10	—	—	—	340	57.0	12-123

**Figure 1:**
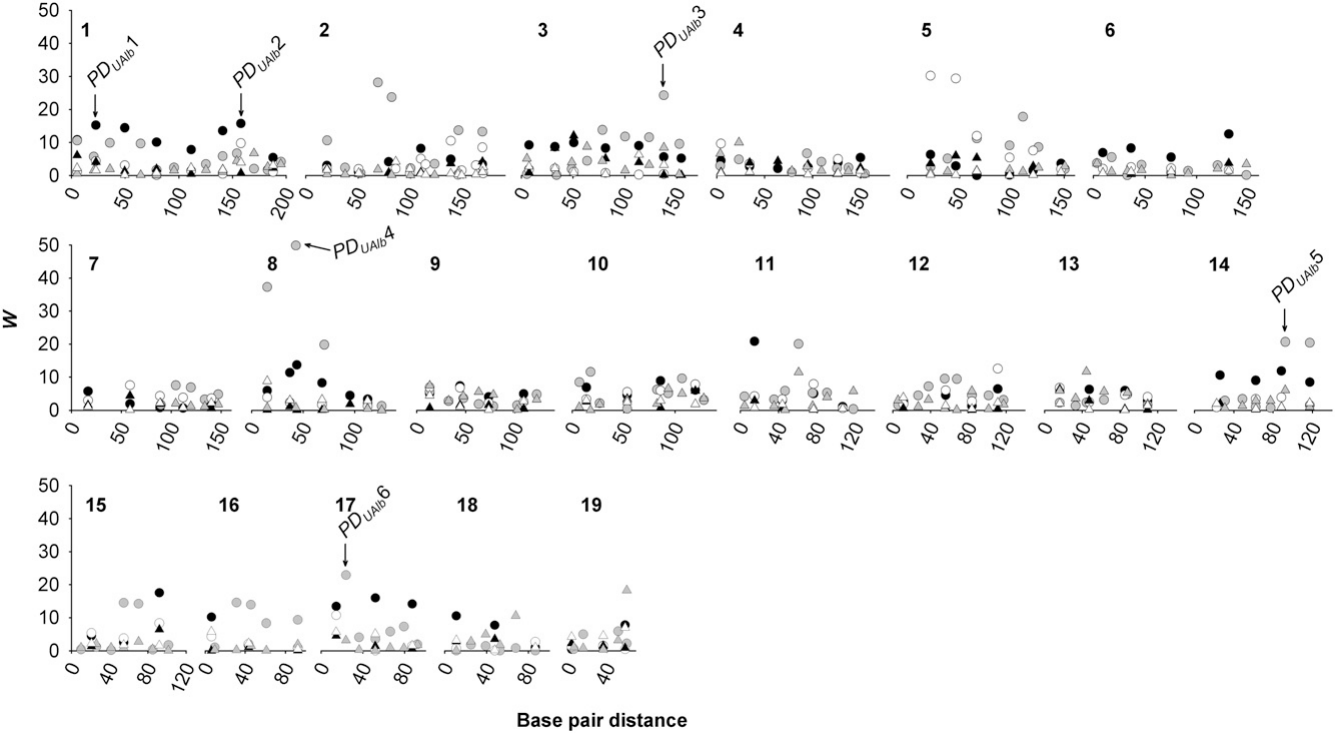
Association of phenotypic dispersion in urinary albumin ($PD_{UAlb}$; circles) and urinary creatinine ($PD_{UCrea}$; triangles) with marker genotype by chromosome in a) a cohort of 383 male F_2_ C57BL/6J × A/J house mice (*Mus musculus*) (Doorenbos *et al.* A) (solid symbols), b) a cohort of 207 male F_2_ C57BL/6J *ApoE^−/−^* × A/J mice (gray symbols) (Doorenbos *et al.* B) (Doorenbos *et al.* 2008) and c) a cohort of 340 male F_2_ DBA/2J × C57BL/6J mice (white symbols) (Sheehan *et al.* 2007). Significant points with maximal association with $PD_{UAlb}$ are indicated (*i.e.*, ‘$PD_{UAlb}1$’).

**Figure 2:**
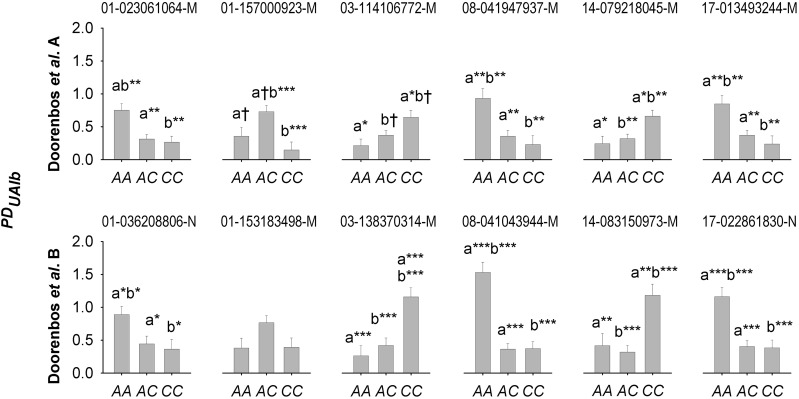
Differences in mean genotypic effects on phenotypic dispersion in urinary albumin ($PD_{UAlb}$) by single nucleotide polymorphism (SNP) genotype for genomic regions syntenically associated with $PD_{UAlb}$ in 383 male F_2_ C57BL/6J × A/J mice (‘Doorenbos *et al.* A’) and 207 male F_2_ C57BL/6J *ApoE^−/−^* × A/J mice (‘Doorenbos *et al.* B’) {Doorenbos, 2008 #25451} (syntenic regions by figure column). SNP marker names are indicated above each graph with the first two digits being chromosomal designation. Significant differences among genotypes for mean *PD* are indicated as *P_Bon_* < 0.10^†^, < 0.05*, < 0.01**, < 0.001***.

Several genomic regions were significantly associated with $PD_{UAlb}$ in both Doorenbos *et al.* cohorts, with highly similar genetic architecture in these mapping groups (Table 2; Figure 1). $PD_{UAlb}$ was associated with SNP genotype over the approximate region of 5-64 MB on Chr 1 in Doorenbos *et al.* A and B, here considered to represent a locus for albuminuria dispersion (‘$PD_{UAlb}1$’) (Table 2; Figure 1). Contrast tests for dominance and additivity indicated that $PD_{UAlb}1$ was partially negative dominant (Lee and Sabapathy 2008) for the C57BL/6J allele, so that C57BL/6J×A/J heterozygotes and C57BL/6J homozygotes had lower $PD_{UAlb}$ than A/J homozygotes (*P_FDR_* < 0.01) (Table 2; Figure 2). $PD_{UAlb}$ was significantly associated with Chr 1 markers in the 141-158 MB range in Doorenbos *et al.* (Table 2; Figure 1). A post-Benjamini correction (*P_FDR_* < 0.05). Contrast tests indicated that this position was overdominant with C57BL/6J×A/J heterozygotes having marginally higher $PD_{UAlb}$ (*P* < 0.1) than C57BL/6J homozygotes and significantly higher $PD_{UAlb}$ (*P* < 0.05) than A/Js (Table 2; Figure 2). In Doorenbos *et al.* B, $PD_{UAlb}$ was only nominally (*P* < 0.05) associated with a marker in this range (SNP 01-153183498-M) but $PD_{UAlb}$ had the same structure at this locus in Doorenbos *et al.* B as in A. In combination, another consensus *PD* locus was considered to exist at this position (‘$PD_{UAlb}2$’).

**Table 2:** Chromosome (Chr), approximate position in megabase-pairs (MB) and centiMorgans (cM; consensus map (Cox *et al.* 2009)), unadjusted nominal significance (*P*), proportion of total *PD_UAlb_* explained by each marker (*r*^2^), and the architecture (‘Form’; A = additive, D+ = positive dominant, D- = negative dominant, OD = overdominant, UD = underdominant) and unadjusted significance of effects (*P_arch_*) for single-nucleotide polymorphisms (SNP) associated with phenotypic dispersion in urinary albumin (*PD_UAlb_*) in 383 male F_2_ C57BL/6J×A/J and 207 male F_2_ C57BL/6J×A/J *ApoE^−/−^* mice (*Mus musculus*) (Doorenbos *et al.* 2008) using quantitative limited (Tobit) models. ‘Locus’ refers to positions detected in the same location in both cohorts. The high-*PD* allele is indicated in brackets under ‘Form’

	Doorenbos *et al.* A						Doorenbos *et al.* B						
Chr	MB (cM)	Marker	*P*	*r*^2^	Form	*P_arch_*	MB (cM)	Marker	*P*	*r*^2^	Form	*P_arch_*	Locus
1	22.6 (8.7)	01-023061064-M	0.0005	0.0401	D- (*A*)	0.0001	35.7 (14.5)	01-036208806-N	0.0072	0.0769	D- (*A*)	0.0027	$PD_{UAlb}1$
					A (*A*)	0.0005					A (*A*)	0.0083	
1	157.9 (67.7)	01-157000923-M	0.0004	0.077	OD	0.0004	154.1 (64.7)	01-153183498-M^1^	0.0351	0.0501	OD	0.0129	$PD_{UAlb}2$
					D- (*A*)	0.0073							
2							68.9 (39.5)	02-069853291-N	< 0.0001		D- (*A*)	<0.0001	
											A (*A*)	<0.0001	
3	113.5 (49.5)	03-114106772-M	0.109	0.0249	A (*B*)	0.0034	137.5 (63.9)	03-138370314-M	< 0.0001	0.203	D- (*B*)	<0.0001	$PD_{UAlb}3$
					D- (*B*)	0.0043					A (*B*)	<0.0001	
5							110.6 (53.4)	05-107871207-M	0.0001		OD	0.0001	
											A (*B*)	0.0178	
6	131.3 (63.4)	06-131929438-M	0.0019		D- (*B*)	0.0007							
					A (*B*)	0.0322							
					UD	0.0059							
8	43.8 (23.9)	08-041947937-M	0.0011	0.0677	D- (*A*)	0.0004	43.0 (23.9)	08-041043944-M	<0.0001	0.240	D- (*A*)	<0.0001	$PD_{UAlb}4$
					A (*A*)	0.0008					A (*A*)	<0.0001	
10	85.9 (42.8)	10-086567143-M	0.0117		D- (*A*)	0.0035	17.9 (7.5)	10-107333522-M	0.0083		D- (*A*)	0.0084	
					A (*A*)	0.0055					UD	0.0187	
11	14.9 (8.6)	11-014984030-M	< 0.0001		OD	<0.0001	60.9 (38.0)	11-061500282-N	< 0.0001		D-	<0.0001	
12							55.0 (22.8)	12-048364436-M	0.0086		A (*B*)	0.0031	
											D+ (*B*)	0.0047	
14	88.5 (44.2)	14-079218045-M	0.0027	0.0331	D- (*B*)	0.0027	92.4 (45.3)	14-083150973-M	< 0.0001	0.159	D- (*B*)	<0.0001	$PD_{UAlb}5$
					A (*B*)	0.0038					A (*B*)	0.0022	
											UD	0.0051	
15	92.1 (14.5)	15-093195380-M	0.0002		A (*A*)	<0.0001	70.3 (32.2)	15-070911071-M	0.0008		D- (*B*)	0.0008	
					D- (*A*)	<0.0001					A (*B*)	0.0085	
											UD	0.0050	
16	6.2 (1.8)	16-005644892-N	0.0061		A (*A*)	0.0021	32.1 (21.4)	16-031026287-C	0.0007		D- (*B*)	0.0003	
					D- (*A*)	0.0021					A (*B*)	0.0010	
17	51.8 (26.8)	17-050794277-N	0.0012	0.0790	D- (*A*)	0.0004	23.8 (12.0)	17-022861830-N	< 0.0001	0.129	D- (*A*)	<0.0001	$PD_{UAlb}6$
					A (*A*)	0.0008					A (*A*)	<0.0001	
18	11.1 (5.7)	18-010953833-N	0.0050		OD	0.0017							

1 The SNP marker 01-153183498-M was only marginally associated with $PD_{UAlb}$ in Doorenbos *et al.* B (*P* < 0.1) but is included here to compare the similarity of its architecture with the presumably syntenic region in Doorenbos *et al.* A.

$PD_{UAlb}$ was significantly associated with SNP genotype in Doorenbos *et al.* A and B over the 50-150 MB range on Chr 3 at the Benjamini threshold (Table 2; Figure 1). Contrast tests at the peak SNPs 03-114106772-M (113.5 MB) in Doorenbos *et al.* A and 03-138370314-M (137.5 MB) in Doorenbos *et al.* B indicated partial negative dominance as for $PD_{UAlb}1$ with significantly higher $PD_{UAlb}$ in C57BL/6J homozygotes than either other genotype in Doorenbos *et al.* B (*P_FDR_* < 0.001), and marginally higher $PD_{UAlb}$ than heterozygotes and significantly higher $PD_{UAlb}$ than A/J homozygotes in Doorenbos *et al.* A (Table 2; Figure 2). This was a wider genomic range than other consensus loci with different ranges of overlap, but based on the significance of marker-$PD_{UAlb}$ associations in these closest markers and the similarity of *PD* means by genotype, it was considered that these results represented a third locus for albuminuria dispersion (‘$PD_{UAlb}3$’).

On Chr 8, $PD_{UAlb}$ was associated with SNP over the 37-43 MB range on Chr 8 in both Doorenbos *et al.* A and B (‘$PD_{UAlb}4$’) (Table 2; Figure 1). Like $PD_{UAlb}1$ and $PD_{UAlb}3$, contrast tests indicated that $PD_{UAlb}4$ was partially negative dominant with the $PD_{UAlb}$ for C57BL/6J homozygotes being higher than either of the other genotypic classes (*P_FDR_* < 0.01) (Table 2; Figure 2). On Chr 14, $PD_{UAlb}$ was significantly associated with genotype over 26.2-88.5 MB in Doorenbos *et al.* A and 92.4-117.4 MB in Doorenbos *et al.* B. (‘$PD_{UAlb}5$’) (Table 2; Figure 1). As most of the other consensus loci, genetic architecture at the SNP 14-079218045-M in Doorenbos *et al.* A and 14-108203728-M in Doorenbos *et al.* had significant negative dominant and additive components (*P* < 0.05) with C57BL/6J homozygotes having higher dispersion than any other genotypic class (*P_FDR_* < 0.05) (Table 2; Figure 2).

Markers in the anterior regions of Chr 17 (peaks at 17-050794277-N (15 MB) in Doorenbos *et al.* A and 17-022861830-N (24 MB) in Doorenbos *et al.* B) were significantly associated with $PD_{UAlb}$ (‘$PD_{UAlb}6$’) (Table 2; Figure 1). As $PD_{UAlb}1$ and $PD_{UAlb}3-5$, this locus also appeared to be partially negative dominant with A/J homozygotes having significantly lower dispersion than C57BL/6J×A/J heterozygotes or A/J homozygotes (*P_FDR_* < 0.01) (Table 2; Figure 2).

**Figure 3:**
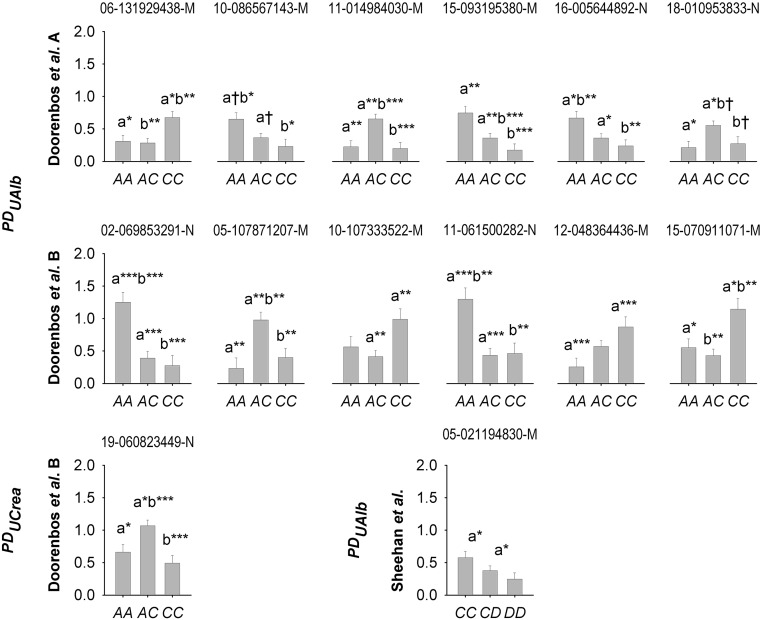
Mean dispersion in urinary albumin ($PD_{UAlb}$) and creatinine ($PD_{UCrea}$) by single nucleotide polymorphism (SNP) genotype for markers detected only in 383 male F_2_ C57BL/6J × A/J mice (‘Doorenbos *et al.* A’) or 207 male F_2_ C57BL/6J *ApoE^−/−^* × A/J mice (‘Doorenbos *et al.* B’) {Doorenbos, 2008 #25451}. SNP markers are indicated above each graph. Significant differences among genotypes for mean *PD* are indicated as *P_Bon_* < 0.10^†^, < 0.05*, < 0.01**, < 0.001***.

There were a number of markers associated with $PD_{UAlb}$ in only one of the two cohorts (Chr 6, 10, 11, 15 and 18 in Doorenbos *et al.* A; Chr 2, 5, 10 and 11 in Doorenbos *et al.* B), largely negative dominant or overdominant (Table 2, Figures 1, 3). Two loci on Chr 15 and 16 were significantly associated with $PD_{UAlb}$ in Doorenbos *et al.* A and B but with contrasting effects in each cohort so that A/J alleles had high random variance in Doorenbos *et al.* A while C57BL/6J alleles had high random variance in Doorenbos *et al.* B (Table 2). In the Sheehan *et al.* C57BL/6J×DBA/2J mice, a single overdominant locus on Chr 5 spanning 22-47 MB was associated with $PD_{UCrea}$, with C57BL/6J×DBA/2J heterozygotes the highest dispersion of the three genotypic classes (*P* < 0.01) (Table 2; Figures 1, 3).

No genomic region was associated with $PD_{BUN}$ at the FDR (*P* > 0.1). A single marker in the anterior end of Chr 19 (SNP 19-060823449-N; 56.5 MB) was significantly associated with $PD_{UCrea}$ (*r*^2^ = 0.183) (Figure 2), here termed $PD_{UCrea}1$ and having overdominant expression for *PD* in C57BL/6J×DBA/2J heterozygotes in Doorenbos *et al.* B (*P* = 0.0002). There was no evidence of this effect in Doorenbos *et al.* A or Sheehan *et al.*; a linked SNP (19-059089086-M) was associated with $PD_{UCrea}$ before FDR correction (Figure 1), appearing dominant (not shown). No locus for dispersion in albuminuria was linked to this position.

### Distribution of genetic architectures

Of all loci significantly associated with *PD* traits in all three cohorts (including dual additive and dominance components for partially dominant loci), 19 were additive, one was high-dominant (heterozygote equal to the high-*PD* homozygote), 20 were negative dominant, five overdominant and four underdominant (Table 2). For those loci with statistical analogs in both Doorenbos *et al.* cohorts, there were ten loci with additive effects, 11 with negative dominance, two overdominants and one underdominant (Table 2).

### C57BL/6J-vs-A/J candidate SNP

SNP between the C57BL/6J and A/J strains linked to dispersion loci occurred in genes affecting cell growth/mitosis/platelet action (*Arid5a*, *Egf*, *Fgl1*, *Fgf20*, *Igfals*, *Itpr3*, *Ogfrl1*, *Plg*, *Rblcc1*, *Rab23*, *Qsox1*), immunology (*Arid5a*, *Lonrf1*, *Msr1*, *Mtus1*, *Phf3*), serine/threonine physiology (*Camk2d*, *Dlc1*, *Dusp4*, *Pkmyt1*, *Prss29*, *Prss30*, *Prss33*, *Prss34*, *Prss40*, *Prss41*, *Smok2b*, *Srrm2*), DNA repair and mitotic checkpoint maintenance (*Eme2*, *Ercc5*. *Mcmdc2*, *Tdrd5*, *Telo2*, *Tex15*, *Tti2*), cellular construction/morphology (*Actr1b*, *Ank2*, *Cep450*, *Col11a1*, *Col5a2*, *Dnah7b*, *Dst*, *Ogfr1*, *Mdga*, *Mtus1*), G-protein coupled receptors (*Fdnc1*, *Fpr3*, *Fpr-rs3*, *Fpr-rs4*, *Fpr-rs6*), calcium physiology (*Bank*, *Dnase112*, *Pcdh9*, *Pkd1*, *Saraf*), gene expression (transcription, splicing, translation) (*Eri1*, *Purg*, *Rbm20*, *Rrp1b*, *Trmt9b*, *Trmt11*, *Trmt13*). Some SNP variants occurred at genes linked to other renal diseases including autosomal dominant polycystic kidney disease (ADPKD) (*Pkd1*) and autosomal recessive polycystic kidney disease (ARPKD) (*Pkhd1*) and cystic fibrosis (*Slc9A3R2*). Two genes (*Ccnf* and *Tbl3*) contained WD-40 domains. There were a variety of SNP in coding sites for vomeronasal genes and in type C2H2 zinc fingers (*Flywch1*, *Wiz1*, *Zgrf1*, *Zfp* proteins) (Table S1).

## Discussion

Six syntenic consensus loci for albuminuria dispersion were detected in Doorenbos *et al.* A and B on Chr 1 (5-64 megabases (MB), $PD_{UAlb}1$; 141-158 MB, $PD_{UAlb}2$), Chr 3 (∼113 MB, $PD_{UAlb}3$), Chr 8 (37-68 MB, $PD_{UAlb}4$), Chr 14 (92-117 MB, $PD_{UAlb}5$) and Chr 17 (14-24 MB, $PD_{UAlb}6$), all unlinked to conventional albuminuria loci. Each syntenic locus in Doorenbos *et al.* had the same genetic architecture in both cohorts, which strongly implies validation of these positions. A single locus for $PD_{UAlb}$ was detected in Sheehan *et al.* on Chr 5 (22.3 – 46.5 MB) with no syntenic effect in either other group. There is significant variance in the onset of albuminuria and CKD (Moranne *et al.* 2009) and the number of independent dispersion loci in this work suggests that albuminuria distributions may be largely determined by a number of independent arrays of genes with randomizing effects on disease onset and progression. The mechanics of dispersion loci could range from ephemeral physiological ‘twitches’ to randomization in the progress of long-term biological insult ranging from the unaffected state to the disease state *vs.* retention of the unaffected status; dispersion in albuminuria, with attendant morphological changes (glomerular damage and inflammation with subsequent podocyte damage from infection, self-response or complement thrombosis) (Doorenbos *et al.* 2008; Coto *et al.* 2013; Regal *et al.* 2018). Only a single locus was detected for dispersion in creatinine; this sole finding against the larger number of loci for albuminuria may reflect more constant creatinine expression as a baseline estimator of kidney throughput (Stevens *et al.* 2013). C57BL/6J *vs.* A/J SNP variants linked to these loci included polymorphisms at various transposable element regulators, respiratory electron chain genes, G-protein coupled N-formyl peptide receptors, vomeronasal genes, serum calcium regulators, complement receptors, signal transducers, and candidates of autosomal dominant (*Pkd1*) and recessive (*Pkhd1*) polycystic kidney disease (Bergmann 2015; Ghata and Cowley 2017) and cystic fibrosis (*Slc9A3R2*) simultaneously mitigates the effects of the cystic fibrosis transmembrane conductance (CFTR) reducing renal cyst growth via proteostasis and reduces resting intracellular Ca^2+^ (Yanda *et al.* 2018). Various SNP occurred in serine/threonine-enriched proteins, which have been associated with loci linked to the coefficient of variation (CV) in total RNA production (Perry, unpublished results), diabetes severity/onset (G. M. L. Perry, unpublished results) and diabetic plasma traits (Brown 2018; G. M. L. Perry, unpublished results). Two genes (*Ccnf*, *Tbl3*) had WD-40 domains (Schapira *et al.* 2017); SNP in WD-40 domains were also associated with random variation in urinary calcium in a human cohort (n = 1210) (Perry *et al.* 2013).

*PD* loci accounted for smaller proportions of randomized variance in Doorenbos *et al.* A (3–8%) than B (5–24%); this may have been due to sample size, the Beavis effect (Beavis 1998) and/or liberating effects of the *Apoe* KO on residual variance in the latter. The removal of mediating factors like *Apoe* might result in increasingly unstable physiological architecture so that downstream systems might also be subject to increasing dispersion, although the mechanics of such an effect would depend on the nature of the physiological pathway. *Apoe*^−/−^ mice have a wide range in nephropathic outcome (Wen *et al.* 2002; Buzello *et al.* 2004). Loci detected in only a single cohort might be related to this dispersive mediation. The genetic architecture in $PD_{UAlb}$ appeared to be inverted between Doorenbos *et al.* A and B for loci on Chr 15 and 16, so that *Apoe* might alter the tendency to dispersion within genotypes.

A strong majority of dispersive loci were partially negative dominant, with contrasts including additive and negative dominant components. This is similar to a recent survey of *PD* for diabetes-related serum traits (high- and low-density lipoproteins, general cholesterol, triglycerides) in eight intercross and backcross mouse cohorts in which most *PD* loci were also negative dominant (Brown 2018). Not all dispersive genetic variance has this expression (Perry *et al.* 2013; G. M. L. Perry, unpublished results) but negative dominance—essentially recessivity for high dispersion where genetic physiological randomization is suppressed by single normalizing alleles which promote constant or stable gene activity—might be a frequent feature of this phenomenon. This propensity to suppression of randomizing variance might thus mean that ‘recessive’ high-*PD* genotypes are essentially detrimental as in other recessive systems (Charlesworth 2009), although the ecological implications of the phenomenon have not been extensively explored. A primarily recessive architecture for randomizing phenotype could also create additional complications in genetic analysis (Hildebrandt *et al.* 2009) similar to limited recessive penetrance (see Boone *et al.* 2013; Gao *et al.* 2015). This might include dispersive loci that influence the detection of normal genes (*i.e.*, Perry *et al.* 2011). New model builds might need to be created in order to specifically address such systems.

### Creatinine

One locus for $PD_{UCrea}$ was detected on Chr 19 (57 MB) in the C57BL/6J×A/J *ApoE*^−/−^ group. As a product of lean muscle mass, creatinine should be relatively stable, but intraindividual CVs for creatinine approximate 9% (Bingham and Cummings 1985) and the heritability of individual CV in urinary creatinine was significant ($h_{s}^{2}$ = 8.7%) in a three-generation cohort of 949 kidney stone probands and first-degree relatives (Perry *et al.* 2012b). Dispersion effects in fitness or survivorship from creatinine might operate through physiological related to disease state: Gibb *et al.* (1989) found higher between-individual variance in creatinine clearance in diabetic children than non-diabetics.

### Multilocus heterozygosity

Heterozygosity-trait correlations (HTCs) are linked with fitness or other traits in many systems (Willoughby *et al.* 2017; Brambilla *et al.* 2018; Kardos *et al.* 2018) but support for Lernerian genetic homeostasis (1977) in trait randomization has been mixed (Perry *et al.* 2012a; G. M. L. Perry *et al.*, unpublished results). MLH was negatively correlated with dispersion in albuminuria in the Doorenbos *et al.* cohorts but not in Sheehan *et al.* There is evidence for variation in heterozygosity-trait correlations over subpopulations (Brock *et al.* 2015) and across ontogeny (Gillingham *et al.* 2013; Annavi *et al.* 2014), due to contextual variance in selection gradients. This implies some tractable, functional variability in HTCs, but the cohorts used here were lab-reared under no known selective pressure, suggesting that differences in MLH correlation were endogenous in origin. Some work indicates that HTCs at specific regions are more important than total individual heterozygosity (Rodríguez-Quilón *et al.* 2015) so that genetic differences between strains might be expected to generate differences in both HTCs and MLH-*PD* correlations. Differences in heterozygosity for specific regions enriched for immunological or other functional groups (*i.e.*, the MHC complex on human Chr 6 and mouse Chr 17 (cytoband B-C)) might, for example, be key to variation in this effect.

### Conclusions

The incidence of heritable dispersion appears to be growing (SanCristobal-Gaudy *et al.* 1998; Perry *et al.* 2003; Sorensen and Waagepetersen 2003; Hill and Zhang 2004; Ordas *et al.* 2008; Hill and Mulder 2010; Perry *et al.* 2012a; Rönnegård and Valdar 2012; Wang *et al.* 2014). Analytically, this represents an enormous potential area of genetic interest: dispersive systems, themselves a series of random risk factors invisible to conventional analysis, could render critical elements of genetic control conventionally undetectable (Perry *et al.* 2011) or mask major elements of trait distributions from genetic decomposition of architecture. High similarity of position and effect for albuminuria dispersion markers across the two cohorts strongly supports the existence of dispersion loci underlying the effect, but there was little evidence that the basis of the effect was in general physiological systems like transcription regulation or splicing. A genetic architecture ranging from negative dominance to additivity (Brown 2018) indicates that high heritable randomization values tend to be recessive so that stable expression is ‘rescued’ by a single normalizing allele. Randomization in signs or elements of disease physiology such as albuminuria might be particularly unfit, generally.

Additionally, the evolutionary consequences of such systems could be profound: dispersion loci could create ‘fuzzy’ surfaces on fitness landscapes, permitting individuals or subpopulations to transit between local adaptive peaks without the risk of intervening saddles, or mitigate competition among siblings by dispersing phenotype in close relatives, or allow single parents to produce an array of heterogenous progeny to exploit new niche space or variable environments. Albuminuria (Syme *et al.* 2006) and creatinuria (Gibb *et al.* 1989) are linked to survivorship so that dispersion in proteinuria may indeed have direct fitness relevance. The elaboration of dispersion in this system and others may provide a powerful insight into the construction of phenotype, elucidating unseen spandrels of distribution in medicine, evolution and agriculture.
